# In Response to Zhang & You

**DOI:** 10.1002/acm2.70821

**Published:** 2026-10-09

**Authors:** Aika Hayashi, Yasukata Takahashi, Naoko Sanuki, Yasufumi Yamashita, Yoshitaka Suzuki, Kanae Matsuura, Hiroe Muto

**Affiliations:** ^1^ Graduate School of Medical Science Suzuka University of Medical Science Suzuka Mie Japan; ^2^ Department of Central Radiology Yokkaichi Municipal Hospital Yokkaichi Mie Japan; ^3^ Department of Radiology Keio University School of Medicine Shinjuku‐ku Tokyo Japan; ^4^ Department of Radiology Yokkaichi Municipal Hospital Yokkaichi Mie Japan; ^5^ Department of Radiology Mie University Hospital Tsu Mie Japan; ^6^ Department of Radiological Technology, Faculty of Health Science Suzuka University of Medical Science Suzuka Mie Japan; ^7^ Center for Fundamental Education of Health Professions Suzuka University of Medical Science Suzuka Mie Japan

Dear Editor‐in‐Chief,

1

We read with great interest and appreciate the thoughtful, constructive comments on our recently published article, “Impact of MRI slice thickness and fiducial marker orientation on geometric accuracy in multi‐echo GRE imaging for prostate radiotherapy planning”.^1^ We thank the correspondents for their detailed analysis and for supporting our primary finding that a thinner (1.0 mm) MRI slice protocol significantly reduces marker‐length measurement error.

The correspondents raise several crucial points about the distinction between “marker‐length measurement error” and “true three‐dimensional localization/displacement error.” We fully agree with their scientific reasoning and appreciate this opportunity to clarify and refine our interpretations.

First, we acknowledge the limitation that our primary metric (the difference between the physically verified 10.0‐mm GFM length and the Euclidean distance calculated from coordinates identified on the treatment planning system) measures marker‐length distortion rather than translational displacement of the marker's centroid or individual tips relative to an independent spatial reference.^1^ As the correspondents rightly point out, symmetric shifts in identified coordinates or isotropic extension of the signal void could affect length measurements without changing the true marker center, and vice versa. Although we acknowledged this intrinsic limitation in the Discussion section of our article, we admit that our use of the term “localization accuracy” in the Abstract and Conclusion was overgeneralized. We agree that it is more accurate to describe this advantage as “reduced marker‐length measurement error” until localization is validated against an independent spatial coordinate system.

Second, regarding the 1.0‐mm slice protocol, we agree that while the overall mean measurement error was sub‐millimeter (0.96 mm; 95% CI, 0.70–1.22 mm), the specific mean errors in 7 of the 12 orientations did slightly exceed 1.0 mm (ranging from 1.04–1.42 mm). The statement that sub‐millimeter accuracy was “maintained across all orientations” was indeed an oversimplification of our tabular data. We appreciate this clarification and agree that the thinner slice protocol minimizes overall measurement error but does not guarantee sub‐millimeter precision at every individual orientation angle.

Third, we concur with the feedback regarding the Van Herk margin simulation.^2^ Our simulation, which utilized the peak measurement error of 3.10 mm as a hypothetical constant systematic shift (Σ), was a worst‐case theoretical exploration rather than a demonstrated clinical mandate. We agree that treating a 3.10‐mm overestimation of length as an equivalent positional displacement goes beyond our experimental measurements, as signal void extension at both ends may leave the marker center unchanged. We appreciate the recommendation to evaluate CT‐MRI registration and fusion accuracy using established registration‐assessment frameworks (such as AAPM Task Group 132) in future clinical studies.^3^

In summary, we are grateful for these comments, which strengthen the scientific rigor of our work. The constructive discussion initiated by the correspondents highlights the physical and geometric nuances of GFM visualization in multi‐echo GRE sequences. We stand by our core conclusion that utilizing a 1.0‐mm slice thickness is highly desirable to minimize partial‐volume effects and geometric distortions in clinical planning,^1^ and we agree that future clinical validation is needed to assess true three‐dimensional localization and CT‐MRI registration accuracy.

Sincerely,

## AUTHOR CONTRIBUTIONS

All authors contributed to the preparation, critical review, and approval of the final version of this response letter.

## CONFLICT OF INTEREST STATEMENT

The authors have no relevant conflicts of interest to disclose.

## ETHICS STATEMENT

This response letter does not involve human participants or animal subjects and therefore did not require ethical approval.

## Data Availability

No new data were generated for this response letter.
